# Prolonged Laccase Production by a Cold and pH Tolerant Strain of *Penicillium pinophilum* (MCC 1049) Isolated from a Low Temperature Environment

**DOI:** 10.1155/2014/120708

**Published:** 2014-03-09

**Authors:** Kusum Dhakar, Rahul Jain, Sushma Tamta, Anita Pandey

**Affiliations:** ^1^Biotechnological Applications, G. B. Pant Institute of Himalayan Environment and Development, Kosi-Katarmal, Almora, Uttarakhand 263 643, India; ^2^Department of Biotechnology, Bhimtal Campus, Kumaun University, Nainital, Uttarakhand 263 136, India

## Abstract

Production of laccase by a cold and pH tolerant strain of *Penicillium pinophilum* has been investigated under different cultural conditions for up to 35 days of incubation. The fungus was originally isolated from a low temperature environment under mountain ecosystem of Indian Himalaya. The estimations were conducted at 3 temperatures (15, 25, and 35°C), a range of pH (3.5–11.5), and in presence of supplements including carbon and nitrogen sources, vitamins, and antibiotics. Optimum production of laccase was recorded at 25°C (optimum temperature for fungal growth) and 7.5 pH. The production of enzyme was recorded maximum on day 28 (11.6 ± 0.52 U/L) following a slow decline at day 35 of incubation (10.6 ± 0.80 U/L). Fructose and potassium nitrate (0.2%) among nutritional supplements, chloramphenicol (0.1%) among antibiotics, and folic acid (0.1%) among vitamins were found to be the best enhancers for production of laccase. Relatively lower but consistent production of laccase for a longer period is likely to be an ecologically important phenomenon under low temperature environment. Further, enhancement in production of enzyme using various supplements will be useful for its use in specific biotechnological applications.

## 1. Introduction

Lignin, one of the biopolymers on earth, is recalcitrant to decomposition due to its heterogeneity of structure. While basidiomycetous fungi are recognized to be the best degraders of lignin due to their higher efficiency toward mineralization of biopolymers [1]; ascomycetous fungi are also getting attention as lignin degraders from various ecological niches [2]. Genus* Penicillium* has been well recognized for its extensive uses in therapeutics and food industry due to the production of antibiotics, mycotoxins, and other secondary metabolites [3, 4]. In addition, the species of* Penicillium* have also been known for their applications in various environmental aspects, such as plant nutrition, bioremediation, and biodegradation [5–7]. Laccase is an important lignin degrading enzyme, also known for its multiple applications [8]. Enhancement in production of microbial laccase through supplements/inducers is well known [9].

In the low temperature environments under mountain ecosystem, the biogeochemical cycle of carbon is greatly affected by the accumulation and slow degradation of biopolymers. Characterization of psychrotolerants with reference to their biodegrading potential is an important aspect for understanding the ecological dynamics of these environments. In a recent study [10], 25 species of* Penicillium* have been reported to dominate the high altitude soils in low temperature environment under mountain ecosystem of Indian Himalayan Region (IHR). Besides ecological importance, these species have also been studied for their biotechnological applications [11]. It was interesting to record that the activities performed by these organisms are relatively slow but persist for longer period. In a plate-based screening, many of these* Penicillium* species were found to be positive for laccase production. In the present study, one of these representative species,* P. pinophilum*, has been investigated for its efficiency for prolonged laccase production under different physicochemical and nutritional conditions.

## 2. Materials and Methods

### 2.1. The Fungus

The fungus,* Penicillium pinophilum* (MCC 1049), was originally isolated from the soil and collected from a glacial site in IHR. The description of the study site has been reported previously [12–14]. The pure culture was maintained on potato dextrose agar (PDA) slants at 4°C in the microbial culture collection of the laboratory. Fresh culture was grown on PDA at 25°C for 5 days, for each experiment.

### 2.2. Estimation of Laccase

#### 2.2.1. Qualitative Assay

Kirk and Farrell [15] (modified) medium, supplemented with ABTS (2, 2′-azino-bis 3-ethylbenzothiazoline-6-sulphonic acid), was used for screening of ligninolytic enzymes. The medium contained (g/L) the following: 2.0 g malt extract, 2.0 g glucose, 2.0 g NH_4_NO_3_, 0.26 g Na_2_HPO_4_, 0.26 g KH_2_PO_4_, 0.5 g MgSO_4_ (7H_2_O), 0.01 g CuSO_4_ (5H_2_O), 0.006 g CaCl_2_ (2H_2_O), 0.005 g FeSO_4_ (7H_2_O), 0.0005 g ZnSO_4_ (7H_2_O), 0.00002 g Na_2_MoO_4_, 0.00009 g MnSO_4_·H_2_O, and 0.00007 g H_3_BO_3_. 5 mm disc of 6 days old culture was used for inoculation. ABTS plates were incubated in 5 sets at 4, 9, 15, 25, and 35°C. Ligninolytic efficiency was calculated by using the formula = ABTS zone diameter/Fungal colony diameter ∗ 100.

#### 2.2.2. Quantitative Assay

50 mL of medium (without ABTS) was prepared and sterilized in 250 mL of Erlenmeyer flasks. 5 mm disc of a 6-day-old culture grown on PDA was used for inoculation. Following incubation, culture was filtered with Whatman Number 1 filter paper. Filtrate was used as crude enzyme. Laccase activity was determined by using ABTS at 420 nm (36000 M^−1 ^cm^−1^) [16]. Enzyme activity is defined as 1 *μ*M of ABTS oxidized per min.

### 2.3. Production of Laccase under Different Culture Conditions

#### 2.3.1. Effect of Physicochemical Conditions (Temperature and pH)

For estimation of the production of laccase at different temperatures, fungal broth was incubated at 15, 25, and 35°C. Enzyme activity was determined at 7 days (weekly) interval up to day 35 of incubation. Estimation of the production of laccase at different pH was done by setting the medium between 3.5 and 11.5 pH (with an interval of 2 units) and inoculating with the fungus following incubation at 25°C.

#### 2.3.2. Nutritional Sources

Six carbon sources (glucose, fructose, maltose, sucrose, starch, and cellulose) and 5 nitrogen sources (ammonium nitrate, ammonium sulfate, ammonium ferrous sulfate, potassium nitrate, and urea) at 0.2% (separately) were used as nutritional supplements. Glucose and ammonium nitrate, being the ingredients in the original medium, were considered as control.

#### 2.3.3. Supplements

Antibiotics (ampicillin, tetracycline, chloramphenicol, gentamicin, and carbenicillin) and vitamins (B1, pyridoxine, biotin, nicotinic acid, and folic acid) at 0.1% concentration were added in the medium (separately) at day 5 of incubation.

Laccase activity was determined at weekly interval up to day 35 of incubation, in each set of experiment.

### 2.4. Statistical Analysis

All the experiments were done in triplicate. Standard deviations are shown as error bars in figures. The data has been analysed by two-way repeated ANOVA and Bonferroni post hoc test. The level of significance was calculated at *P* < 0.05 and 0.01.

## 3. Results and Discussion

**Figure 1:**
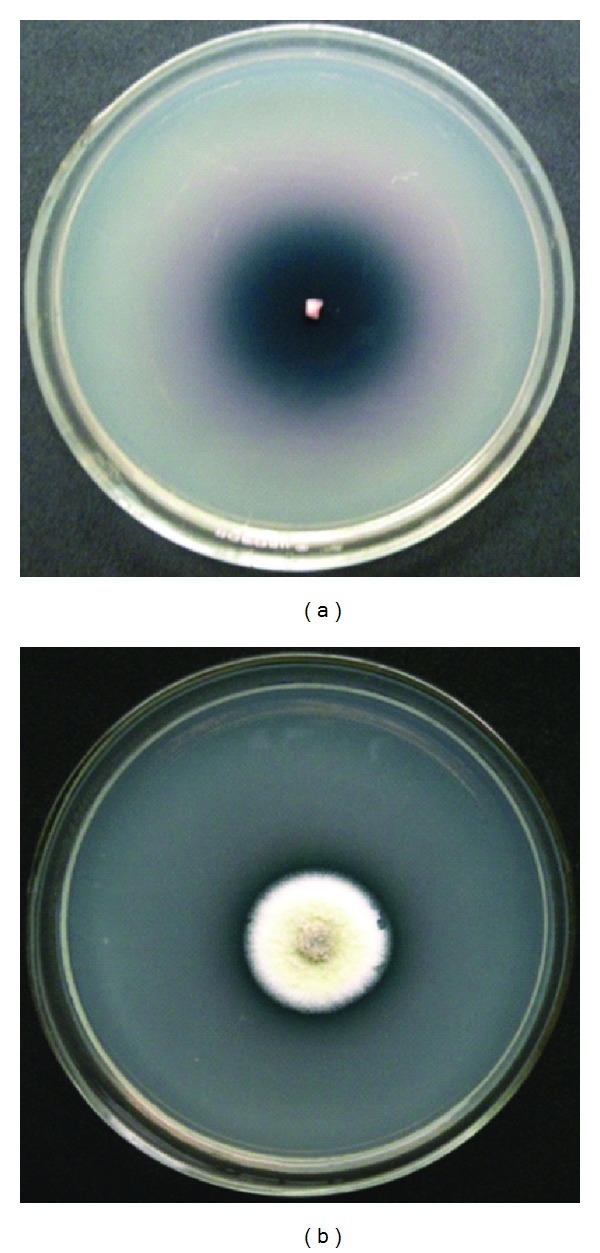
Ligninolytic activity of* P*.* pinophilum* at 4° and 25°C after 15 and 7 days of incubation, respectively.

In qualitative estimations, ligninolytic efficiency was observed increasing with decreasing temperature. Zone to colony diameter ratio was recorded highest at 4°C showing highest efficiency (466.6). The efficiency decreased with the increase in temperature, recorded between 220.0 (9°C) and 17.6 (25°C) (Figure 1). At 35°C the fungus gave moderate growth with minimized activity without forming any clear zone around the colony (Table 1). Production of higher amount of laccase with minimum biomass has been observed in other laccase producing fungi as well [17]. Research findings on similar lines, supporting the production of secondary metabolites in higher amounts by a range of fungi under suboptimal conditions, have been noted as an important phenomenon of ecological as well as biotechnological relevance [11]. Consistent production of secondary metabolites for a longer period maintaining minimized fungal growth, as observed in the present study, can be attributed to the strategies possessed by the organisms for their survival under extreme environments.

**Figure 2:**
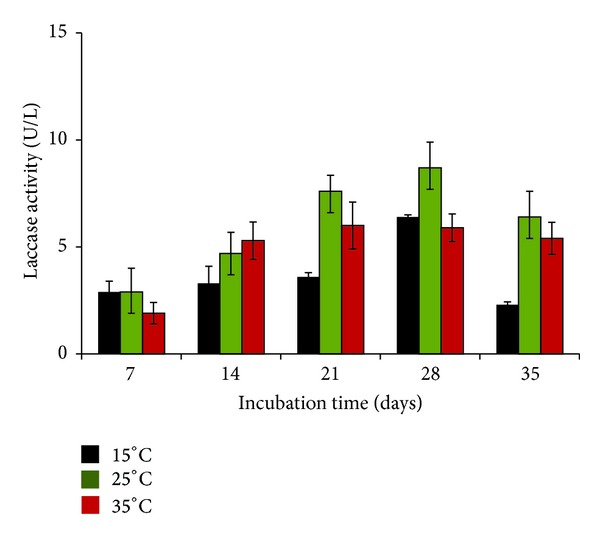
Effect of temperature on laccase production by* P*.* pinophilum for* up to 35 days of incubation.

In quantitative estimations that were carried out at 3 temperatures (15, 25, and 35°C) up to 35 days, the laccase activity was recorded with increasing values up to day 28 of incubation (8.7 ± 0.3 U/L) at 25°C (optimal growth temperature). This activity was higher in comparison to the values recorded at 15° as well as 35°C, being statistically significant (*P* < 0.01) at 15°C (Figure 2). In previous studies, optimum temperature for production of laccase enzymes has been reported under mesophilic range [18–20]. Zadrazil et al. [21] reported that higher temperature (>30°C) was not suitable for production of laccase. The earlier findings on laccase producing organisms have been based on fungi from warmer locations. In these studies the estimations were performed between 1 and 3 weeks probably due to the decline in the activity during this period [18, 22, 23].

**Figure 3:**
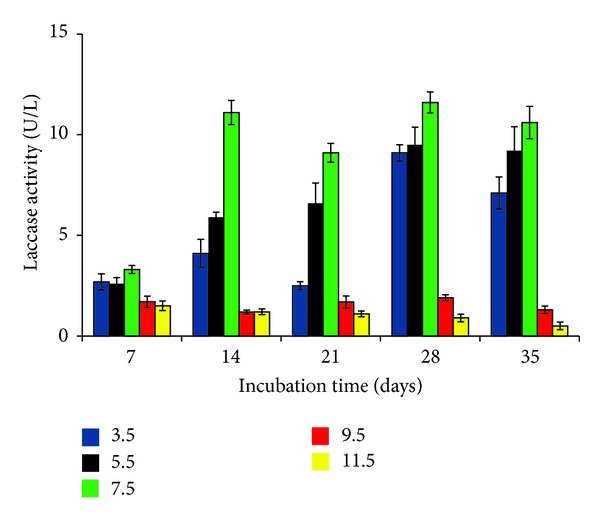
Effect of pH on laccase production by* P*.* pinophilum for* up to 35 days of incubation.

Laccase activity was recorded at wide range of pH from acidic to alkaline (between 3.5 and 11.5), being optimum at 7.5 pH (11.6 ± 0.5 U/L). The optimum activity recorded in this case was statistically significant (*P* < 0.01) showing stability between 5.5 and 7.5 pH with maximum biomass production (Figure 3). Maximum production of laccase at 7.5 pH has also been reported by Janusz et al. [24]. In general, the production of laccase is favoured by acidic pH [20]. Variation in pH is known to affect the 3D structure of the enzyme influencing the enzyme activity [25]. While the fungus under study has been reported to survive at a wide range of pH, that is, 1.5 to 14.0 [10], it also produced laccase at a wide pH range. This is likely to be another character possessed by the fungus for its survival under extreme environment.

**Figure 4:**
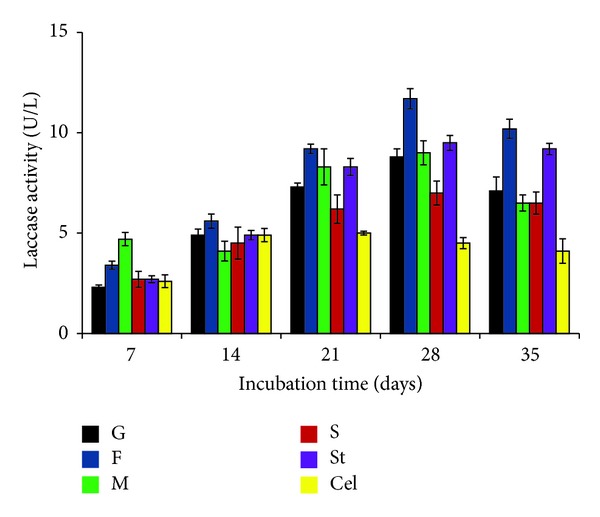
Effect of carbon sources on laccase production by* P*.* pinophilum for* up to 35 days of incubation. G: glucose (control), F: fructose, M: maltose, S: sucrose, St: starch, and Cel: cellulose.

**Figure 5:**
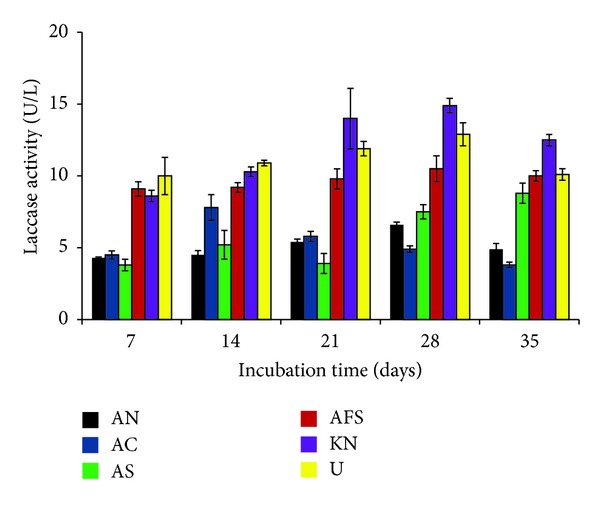
Effect of nitrogen sources on laccase production by* P*.* pinophilum* for up to 35 days of incubation. AN: ammonium nitrate (control), AC: ammonium chloride, AS: ammonium sulfate, AFS: ammonium ferrous sulfate, KN: potassium nitrate, and U: urea.

All the supplements used in the study showed variable effects on production of laccase. Among carbon sources, fructose (0.2%) was found to be the best for production of enzyme. The highest laccase production (11.7 ± 0.5 U/L) (*P* < 0.01) in presence of fructose was recorded at day 28 of incubation. Next to fructose, maltose and starch, respectively, were found to be efficient enhancers for production of laccase (Figure 4). Among nitrogen sources, all the sources significantly (*P* < 0.05) enhanced the activity of laccase, being the highest (14.9 ± 0.5 U/l) in case of potassium nitrate at day 28 of incubation. Ammonium chloride that was found to increase the laccase activity up to day 14 became inhibitory at later stage (*P* < 0.05). Urea supported production of laccase up to some extent. Potassium nitrate, when used at 0.2% concentration, enhanced the laccase activity up to 14.9 ± 0.5 U/L (Figure 5).

In general, glucose is known for optimum production of fungal biomass and enzymes [26], while in the present study fructose was observed as the best carbon source. Variable effects of carbon and nitrogen sources on laccase have been studied by various workers [23, 27–30]. A recent study [31] reported more than 10-fold increase in laccase production in the presence of galactose by* Penicillium* sp. High nitrogen concentration is known to limit the production of laccase. Potassium nitrate, beyond 0.2% concentration, was observed as a moderate enhancer of laccase production in the present study.

**Figure 6:**
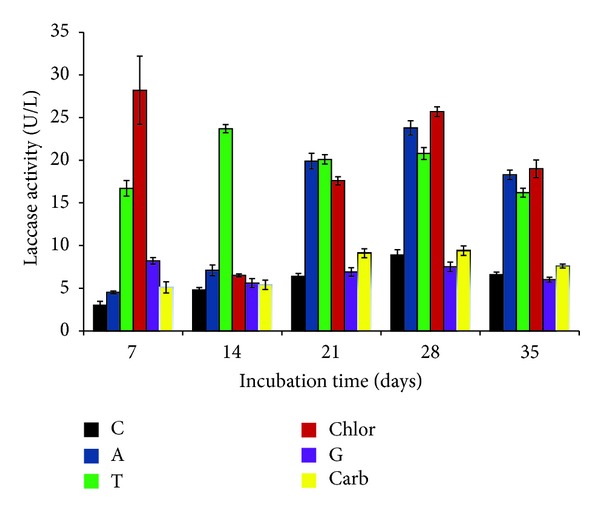
Effect of antibiotics on laccase production by* P*.* pinophilum* for up to 35 days of incubation. C: control, A: ampicillin, T: tetracycline, Chlor: chloramphenicol, G: gentamicin, and Carb: carbenicillin.

**Figure 7:**
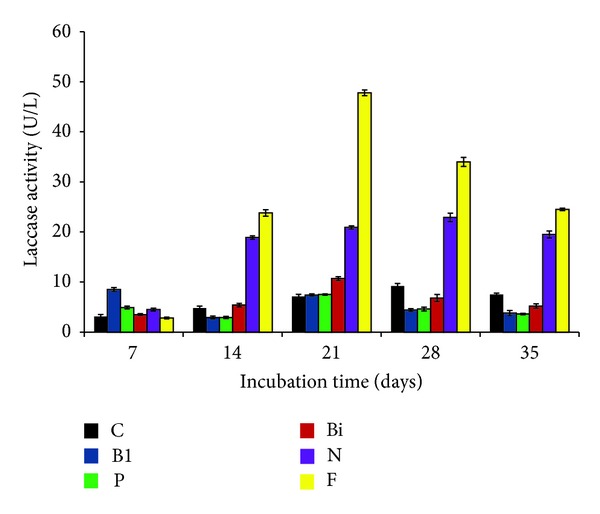
Effect of vitamins on laccase production by* P*.* pinophilum* for up to 35 days of incubation. C: control, B1: vitamin B1, P: pyridoxine, Bi: biotin, N: nicotinic acid, and F: folic acid.

Laccase production was also significantly influenced by the addition of antibiotics or vitamins. Among five antibiotics, chloramphenicol was found to be the best enhancer of laccase (28.2 ± 4.0 U/L) at day 7 of incubation (*P* < 0.01). The similar enhanced effect of this antibiotic was also recorded at day 28 of incubation. Tetracycline and ampicillin also showed the enhancing effect on laccase production during day 21 to day 28 of incubation (*P* < 0.01) (Figure 6). Addition of vitamins (0.1%) revealed varied effects on laccase production (Figure 7). Among 5 vitamins, folic acid was the best laccase enhancer, being statistically significant in all the cases (*P* < 0.01). Folic acid showed enhanced effect between day 14 and day 35 (47.5 ± 0.57 U/L) of incubation. Nicotinic acid was found to be the second potent enhancer of laccase, producing laccase 22.9 ± 0.89 U/L at day 28 of incubation. There have been very few studies related to the effect of antibiotics and vitamins on laccase production. Enhancing effect of antibiotics on laccase production from* Cyathus bulleri* and* Pycnoporus cinnabarinus* has been reported by Dhawan et al. [32].

Species of* Aspergillus*,* Paecilomyces*,* Penicillium*, and* Trichoderma* have been reported to colonize the low temperature environments under mountain ecosystem of IHR [6, 33–37]. Research on the psychrotolerants with reference to their diversity, ecological and biotechnological applications, and adaptations is scanty and needs focused attention in future research.

## 4. Conclusion

Fungi, in general, are preferred sources for biodegradation by virtue of their ability to produce versatile enzymes. While Basidiomycetes are the best known degraders, Ascomycetes, such as species of* Aspergillus* and* Penicillium*, are getting attention as degraders, mainly in low temperature environments. In the present study, a cold and pH tolerant species of* Penicillium*, that is,* P. pinophilum*, exhibited its ability to produce laccase with varying response at a range of temperature and pH for longer period. These characteristics make the fungal strain more efficient for the degradation in the extreme conditions. Further, enhanced enzyme production by some of the nutritional sources and supplements will be important in optimization of the growth conditions for using the fungus for biotechnological applications.

## Figures and Tables

**Table 1:** Ligninolytic efficiency of fungus at different temperature.

Temperature (°C)	Incubation time (days)	ABTS zone Dia. (mm) (ZD)	Fungal colony Dia. (mm) (CD)	Ligninolytic efficiency = (ZD/CD)∗100
4	21	14	3	466.6
9	21	11	5	220.0
15	14	8.0	10.0	80.0
25	07	3.0	17.0	17.6
35	07	0.0	15.0	0.0

Dia.: diameter.
